# Natural Sleep

**Published:** 1886-09

**Authors:** 


### HALL'S
### JOURNAL OF HEALTH

VOL. 33. SEPTEMBER, 1886. No. 9

### *NATURAL SLEEP.*

Both mind and body require for their healthy action, regular periods of rest, and nature has wisely ordained that during these periods the curtain shall be let down, and those objects which engage our attention during the ordinary intervals of labor, as effectually shut out of view as if they belonged to another world. Thus, the wasted energies are given a chance to recuperate and refresh themselves, with every revolution of the earth. Nature wastes no effort upon non-essentials; if this were not a necessary provision it would not be. Nothing is made in vain. Food and air and water are no more essential to life and health than is

"Nature's sweet restorer, balmy sleep."

There is indeed no single cause which so prostrates the vital forces and destroys one's mastery of himself, as continued loss of sleep, which if too long endured, the intellectual faculties relax their hold and the body perishes. Hence the importance of a subject which has not escaped the attention of able philosophic minds of all periods.

The late Robert Dale Owen, in treating of the subject of "Sleep in General," expresses himself in this wise:

"If we sit down to make clear to ourselves what is, and what is not, marvelous—to define with precision, the *wonderful*—we may find the task much more difficult than we apprehend. The extraordinary usually surprises us the most; the ordinary may not only be far more worthy of our attention, but far more inexplicable also.

"We are accustomed to call things *natural* if they come constantly under our observation, and to imagine that that single word embodies a sufficient explanation of them. Yet there are daily wonders, familiar household marvels, which, if they were *not* familiar, if they were not of daily occurrence, would not only excite our utmost astonishment, but would also, beyond question, provoke our incredulity.

"Every night, unless disease or strong excitement interpose, we become ourselves the subjects of a phenomenon which, if it occurred but once in a century, we should regard—if we believed it at all—as the mystery of mysteries. Every night, if blessed with health and tranquility, we pass in an unconscious moment the threshold of material existence; entering another world where we see, but not with our eyes; where we hear, when our ears convey no perception; in which we speak, in which we are spoken to, though no sound pass our lips or reach our organs of hearing."

There is an exquisite Fairy Tale by the Brothers Grimm, which relates how, according to a weird prediction, a lordly castle and a kingly household were suddenly transfixed and spell-bound by sleep, which continued unremittingly for a hundred years, during which everything remained in *statu quo*.

The castle itself, its ample grounds and internal and external decorations and embellishments—its animals and fowls, from the horses in the stable, the hunting dogs in the court, and the doves on the roof; its courtiers and men and maids in waiting, including chiefs and subalterns of high and low estate; even the king himself, and the queen, and the youthful princess, in the very bloom of happy girlhood—all, all within the charmed precincts were seized and firmly held in the grasp of the incorrigible visitor. Everything was at a standstill, mute and motionless as the fixed presentment of living scenes on the surface of a sensitive plate.

Now what is this but a picture of sleep, so real and natural, that if we eliminate its one marvel of fancied duration, we are able to perceive merely an everyday occurrence familiar to all alike.

But now, even as we gaze upon it, the picture changes. The morning dawns, the sun rises; it is the very morn which completes the eventless and yet eventful hundred years. What do we see? Instantly and with one accord all eyes are opened, and realizing only a common event, every living thing takes up his single thread of life at the very point where it was stayed a century before. Everybody and everything is astir, all avocations are resumed, all duties proceeded with, as though they had been suspended but for a single night.

One of the happiest conceits of Washington Irving is the pathetic story of Rip Van Winkle, who, after his long sleep, awoke to find himself a stranger in a land familiar to his every step. Time is no laggard, and he who abuses nature's kindliest provisions by over-indulgence must expect to be left far behind and greatly the loser for it. The exactions of nature are order and uniformity. The well-remembered aphorism of Sir Thomas Brown is directly in point: "Half our days we pass in the shadow of the earth, and the brother of death exacteth a third part of our lives." Sleep is indeed one of nature's *exactions*. It is almost her only means of restoring to the system the wasted energies incident to the ordinary pursuits of life, and its recurring periods are none too frequent to meet our physical demands. But as to the requisite hours which should be allotted to sleep, no invariable rule can be laid down alike suited to all persons, as it is well understood that physical needs vary as physical constitutions do. Eight hours is the time indicated by Sir Thomas Brown, and this has been long accepted as the average period.

As a rule young people require more hours of rest than older persons. They are much given to exercise in the open air, the material waste is more rapid, and consequently the demands of nature more urgent and uncompromising.

The inability to habitually indulge in sound, refreshing sleep denotes an unnatural condition, which should be immediately corrected. Oftentimes it is the result of some form of dissipation. Over-work, severe mental strain, with irregular intervals of rest, are abuses which nature sets down to the debit side of the unbalanced sheet; and the day of reckoning, if deferred from time to time, is sure to come at last. A resort to drugs to produce drowsiness is only a counterbalance of evils, with a continual gain at the wrong end of the beam. They lower the life currents, weaken the nervous system, and create appetites as harmful and unnatural as they are difficult to overcome. What, then, is to be done? In some instances, *very little.* In others, *very much. Forsake bad habits and return to good ones.* This is the sum and substance of it all. It is nature's fiat, which the whole of *materia-medica* is powerless to resist. Divide the day into timely periods of avocational labor, healthful exercise and recuperative rest, and see to it that neither infringes upon the other. Avoid excesses of every kind, especially in eating, drinking, and needless exposure to heat or cold, and credit nature with that good health of which indifference and neglect would impoverish you.

